# I8-arachnotocin–an arthropod-derived G protein-biased ligand of the human vasopressin V_2_ receptor

**DOI:** 10.1038/s41598-019-55675-w

**Published:** 2019-12-17

**Authors:** Leopold Duerrauer, Edin Muratspahić, Jasmin Gattringer, Peter Keov, Helen C. Mendel, Kevin D. G. Pfleger, Markus Muttenthaler, Christian W. Gruber

**Affiliations:** 1 Institute of Pharmacology, Center for Pharmacology and Physiology, Medical University of Vienna, Vienna, Austria; 2 School of Biomedical Sciences, Faculty of Medicine, The University of Queensland, Brisbane, Queensland Australia; 3 Institute for Molecular Bioscience, The University of Queensland, Brisbane, Queensland Australia; 4 Institute of Biological Chemistry, Faculty of Chemistry, University of Vienna, Vienna, Austria; 5 Monash Institute of Pharmaceutical Sciences, Monash University, Parkville, Victoria Australia; 6 Centre for Medical Research, The University of Western Australia and Harry Perkins Institute of Medical Research, Nedlands, Western Australia Australia

**Keywords:** Receptor pharmacology, Peptides

## Abstract

The neuropeptides oxytocin (OT) and vasopressin (VP) and their G protein-coupled receptors OTR, V_1a_R, V_1b_R, and V_2_R form an important and widely-distributed neuroendocrine signaling system. In mammals, this signaling system regulates water homeostasis, blood pressure, reproduction, as well as social behaviors such as pair bonding, trust and aggression. There exists high demand for ligands with differing pharmacological profiles to study the physiological and pathological functions of the individual receptor subtypes. Here, we present the pharmacological characterization of an arthropod (*Metaseiulus occidentalis*) OT/VP-like nonapeptide across the human OT/VP receptors. I8-arachnotocin is a full agonist with respect to second messenger signaling at human V_2_R (EC_50_ 34 nM) and V_1b_R (EC_50_ 1.2 µM), a partial agonist at OTR (EC_50_ 790 nM), and a competitive antagonist at V_1a_R [pA_2_ 6.25 (558 nM)]. Intriguingly, I8-arachnotocin activated the Gα_s_ pathway of V_2_R without recruiting either β-arrestin-1 or β-arrestin-2. I8-arachnotocin might thus be a novel pharmacological tool to study the (patho)physiological relevance of β-arrestin-1 or -2 recruitment to the V_2_R. These findings furthermore highlight arthropods as a novel, vast and untapped source for the discovery of novel pharmacological probes and potential drug leads targeting neurohormone receptors.

## Introduction

Oxytocin (OT) and vasopressin (VP) are prototypical neuropeptides that together with their G protein-coupled receptors (GPCRs), OTR, V_1a_R, V_1b_R, and V_2_R, form a versatile neuroendocrine signaling system in humans. Peripherally, OT is important in the regulation of labor^1^ and milk let-down^2^, while VP is crucial for water homeostasis and vasoconstriction^3,4^. Centrally, OT and VP influence different behaviors, such as empathy^5^, social recognition^2,6–8^, sexual arousal^2,6–8^, attachment^9^, parental care^10^, and anxiety-related behavior^11^. Consequently, its dysregulation is associated with a wide range of disorders, including post-partum complications, cardiovascular diseases, diabetes insipidus and dysmenorrhea as well as social anxiety disorders, autism, schizophrenia, Prader-Willi syndrome and depression^2,12–18^.

**Figure 1:**
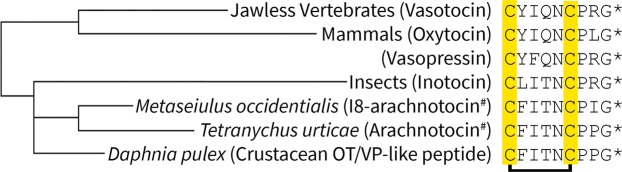
Phylogenetic relationship and molecular sequence of OT/VP-like neuropeptides. Taxonomic groups and neuropeptide names (in brackets). Conserved cysteines are highlighted in yellow, and the disulfide bond between Cys_1_ and Cys_6_ is indicated. *C-terminus amidated; #Renamed to arachnotocin to simplify nomenclature. Analogous to the existing phylum nomenclature, we refer to the mite-derived peptide by I8-arachnotocin, to distinguish it from arachnotocin (also referred to as crustacean OT/VP-like peptide in the past)^20,21^.

OT and VP differ only in two amino acids and their receptors share ~80% binding site sequence homology^19^, which results in OT and VP being unselective and able to activate all four receptors. Consequently, neuroscientists are looking for a repertoire of ligands with different pharmacological profiles to study the (patho)physiology of the individual receptor subtypes of this fundamental signaling system. In an attempt to provide such a pharmacological toolbox, we started to explore different animal species in the search for OT/VP-like ligands that are active on the human receptors, yet better at discriminating signaling between the four OT/VP receptors. Our strategy^14^ relies on the fact that OT/VP-like signaling system is highly conserved and widely distributed across vertebrates, including fish and amphibians, as well as in several invertebrate species such as mollusks, annelids, nematodes, insects, starfish, and hydra^6,14,20^. Its origin can be traced back ~600 million years to the closely-related ancestral vertebrate nonapeptide vasotocin and similar invertebrate nonapeptides (Fig. 1)^20,21^. Our strategy has already yielded several important pharmacological probes, including a selective human V_1a_R antagonist based on an OT/VP-like peptide isolated from the black garden ant *Lasius niger*^19^, a selective antagonist at the human V_1a_R antagonist, based on an OT/VP-like peptide from the venom of the marine predatory cone snail^22^, and a selective human OTR agonist based from an OT/VP-like sequence identified in a cyclotide from the plant *Oldenlandia affinis*^23^.

In this work, we report the chemical synthesis and pharmacological characterization of I8-arachnotocin across the four human OT/VP receptors using second messenger quantification as well as β-arrestin-1 and -2 recruitment assays in cells heterologously expressing the individual receptor subtypes OTR, V_1a_R, V_1b_R, and V_2_R.

## Materials and Methods

### Peptide synthesis

I8-arachnotocin was produced by solid phase peptide synthesis (SPPS) using fluorenylmethyloxycarbonyl (Fmoc) chemistry, purified and analyzed using methods previously described^19^. Briefly, the peptide was synthesized on a Rink amide 4-methylbenzyhydrylamine (MBHA) resin (Auspep, Australia) using 4-fold excess of protected amino acids, 4 equivalents of hexafluorophosphate benzotriazole tetramethyl uranium (HBTU) (Iris Biotech, Germany) and N,N-diisopropylethylamine (DIPEA) (Auspep, Australia) in dimethylformamide (DMF) (Auspep, Australia) and a coupling time of 15 min. The peptide was cleaved from the resin and the side chain protecting groups were removed by treatment with trifluoroacetic acid (TFA) (Auspep, Australia): triisopropylsilane (TIPS) (Sigma Aldrich, Australia): ethandithiol (EDT) (Sigma Aldrich, Australia): H_2_O (90:2.5:2.5:5) for 2 h. The crude peptide was folded for 24 h at 100 μM at 25°C in 0.1 M NH_4_HCO_3_ (Auspep, Australia), pH 8.2, and purified using a Vydac Protein and Peptide C_18_ preparative column. Analytical HPLC was performed with column heating at 40 °C and detection at 214 nm. The final product was analyzed by LC-MS on an API QSTAR PULSAR from PE Sciex, used in series with Agilent 1100 series HPLC system with a Kromasil C_18_ column at a gradient of 0–40% eluent B (90% acetonitrile, 0.045% trifluoroacetic acid) in 20 min.

### Cell culture and transient receptor expression

All cell culture work was performed with human embryonic kidney cells 293 (HEK293)^24^. Unless otherwise stated, cells were incubated at 37 °C and 5% CO_2_ and grown in Dulbecco’s Modified Eagle’s Medium (DMEM; Thermo Fisher Scientific, Australia and Fisher Scientific, Austria) containing 10% fetal bovine serum (GE Life Sciences, Australia and Sigma-Aldrich, Germany), 50 U/mL penicillin and 50 U/mL streptomycin (Thermo Fisher Scientific, Australia and Sigma-Aldrich, Germany). Transient transfections were performed *via* Lipofectamine 2000 (Thermo Fisher Scientific, Australia) or jetPRIME (Polyplus transfection, France) using 2 µg of pEGFP-N1 plasmid DNA coding for EGFP-tagged human OT/VP receptors^23^ or a combination of 2 µg each of receptor-encoding plasmids and a plasmid coding for β-arrestin-1- or -2-Nluc (subcloned into pcDNA3, with NanoLuc provided under a Limited Use Label License from Promega, Madison, USA).

### Second messenger quantification

Inositol-1-phosphate (IP_1_) accumulation in response to Gα_q_ coupled activation of human OTR, V_1a_R, and V_1b_R were measured using the IP-One Gq assay kit (Cisbio, France). Cells were seeded 4 h after transfection onto 384-well plates at a density of 10,000 cells per well and incubated for 2 days. At the time of the assay, all media was removed and the cells equilibrated to the provided stimulation buffer for 15 min at 37 °C. The cells were stimulated with peptide ligands at varying concentration (10 pM **–** 30 µM) for 1 h at 37 °C. Cyclic adenosine monophosphate (cAMP) accumulation induced by Gα_s_ coupling of V_2_R activation was determined using the LANCE Ultra cAMP detection kit (Perkin Elmer, Waltham, USA). Cells were re-passaged 4 h after transfection at a 1:2 ratio and incubated overnight. The next day, all media was removed, and cells were suspended with cAMP stimulation buffer (5 mM HEPES, 0.5 mM 3-isobutyl-1-methylxanthine, 0.1% bovine serum albumin in Hank’s balanced salt solution, HBSS, pH 7.4) and the cells transferred onto a 384-well plate at a density of 300 cells per well. Stimulation was carried out for 30 min at 25 °C. Second messenger levels were measured by homogenous time-resolved fluorescence resonance energy transfer *via* fluorescence measurement on a Flexstation 3 (Molecular Devices, San Jose, USA) using the ratios 665/620 nm (IP_1_) and 665/615 nm (cAMP) at an excitation wavelength of 340 nm. For antagonist screening, cells were stimulated with the endogenous ligand OT at OTR (50 nM), or VP for V_1a_R (1 nM), V_1b_R (3 nM) and V_2_R (0.5 pM), in presence (1 or 10 µM) and absence of I8-arachnotocin, as well as with 10 µM of the respective endogenous ligand.

Antagonism of I8-arachnotocin at V_1a_R was characterized by Schild regression analysis (as published earlier)^19^. Briefly, several concentration-response curves of the endogenous agonist VP (as described above) were measured in the presence (1 µM, 3 µM and 10 µM) and absence of I8-arachnotocin. The logarithm of the dose-ratio (A′/A-1) was plotted vs. the logarithm of the respective concentration of I8-arachnotocin (B) to obtain the pA2 value.

### β-arrestin-1 and -2 recruitment

Recruitment of β-arrestin-1- and -2 upon receptor stimulation was measured *via* real-time measurement of bioluminescence resonance energy transfer (BRET) between β-arrestin-1/2-luciferase and EGFP-tagged receptors. Cells were co-transfected with β-arrestin-1/2-Nluc and OT/VP receptor encoding plasmids at a ratio of 1:10. At 6 h post-transfection, the cells were transferred onto a white, clear bottom 96-well plate at 50,000 cells/well in phenol-red free DMEM containing 10% fetal bovine serum. The following day, the cells were serum starved for 1 h in phenol-free DMEM. Furimazine (Promega, Madison, USA), diluted 1:50 in HBSS, was added to the cells 5 min prior to monitoring at a 1:1 ratio. Light emissions were measured at 460 nm (Nluc) and 510 nm (EGFP) on a Flexstation 3 (Molecular Devices, San Jose, USA). After establishment of a baseline for 5 min, peptides diluted in HBSS were added and the response measured for 35 min. The ligand-induced BRET signal was calculated as: (emission EGFP_ligand_/emission Nluc_ligand_) − (emission EGFP_HBSS_/emission Nluc_HBSS_). Concentration-response curves were generated from the BRET signal at 5 min after addition of various peptide concentrations (10 pM – 30 µM).

### Immunoblotting for ERK 1/2

Immunoblotting was performed as described previously^25^. Briefly, following an overnight incubation and 16 h starvation HEK293 cells transiently expressing human V_2_R were treated with 1 µM I8-arachnotocin or 1 µM VP prepared in DMEM. Cells were incubated with peptide ligands at 37 °C for indicated periods and 1 mL of chilled 1x phosphate-buffered saline were used to terminate the incubation. After freeze-thaw cycle in liquid nitrogen cells were solubilized in 100 µl of lysis buffer (50 mM HEPES, 0.5% NP-40 substitute, 50 mM glycerol-2-phosphate, 250 mM NaCl, 5 mM EDTA, 2 mM imidazole, 1 mM Na_3_VO_4_, 1 mM hepta-molybdate, pH adjusted to 7.0 with NaOH), freshly added 1 mM PMSF, one cOmplete Mini EDTA-free tablet (Roche) and one PhosSTOP tablet (Roche) and then centrifuged at 10,000 × g for 10 min. The bicinchoninic acid assay (Micro-BCA kit, Pierce) was used to measure total protein content. Phosphorylated and total ERK 1/2 were detected by immunoblotting on the same membrane using the same exposure method with an anti-phospho-p44/42 MAPK antibody (ERK 1/2) (1:1,000; Cell Signaling Technology) and an anti-p44/42 MAPK (ERK 1/2) (1:1,000; Cell Signaling Technology), respectively. Detection and quantification of resulting bands were executed by secondary antibodies (Donkey anti rabbit 680RD and 880RD) and Odyssey Clx (LiCor Biosciences) infrared fluorescent imaging system, respectively.

### Data analysis

All data were analyzed using GraphPad Prism (GraphPad Software, San Diego) and all graphs were normalized to the activity of OT/VP above baseline. Concentration response curves were fitted to three-parameter non-linear regression curves with a bottom constrained to zero, a slope of one and sigmoidal shape at logarithmic scale to derive estimates of potency (EC_50_) and maximum efficacy (E_max_). Concentration response curves for Schild regression analysis^26^ were additionally constrained to a top value of one hundred. All data were presented as mean ± SEM of at least three independent experiments (unless otherwise stated) conducted in triplicate.

### Ethics Statement

The study presented in this manuscript did not involve human or animal subjects.

## Results

### I8-arachnotocin is an agonist at human OTR, V_1b_R and V_2_R

**Figure 2:**
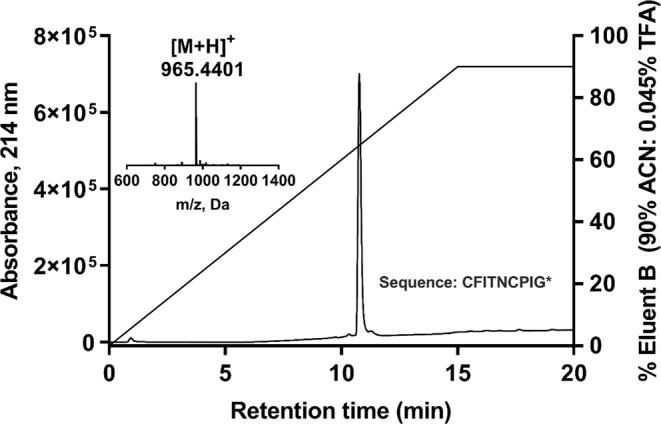
Quality of synthetic I8-arachnotocin. Analytical RP-HPLC of the purified I8-arachnotocin (purity > 95%). Inset: high resolution MS of the product with the observed molecular weight of 964.44 Da (theoretical: 964.44 Da). ACN = acetonitrile; TFA = trifluoroacetic acid; *C-terminus amidated.

**Figure 3:**
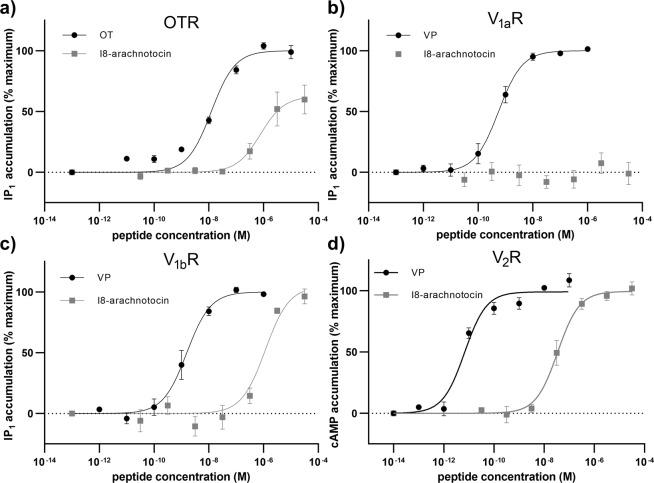
I8-arachnotocin is an agonist at human V_2_R, V_1b_R, and OTR. Concentration-dependent accumulation of second messengers (cAMP and IP_1_) after receptor stimulation of **(a)** OTR, **(b)** V_1a_R, **(c)** V_1b_R and **(d)** V_2_R with I8-arachnotocin (30 pM – 30 µM). Results were normalized to accumulation of IP_1_ and cAMP above baseline and maximal activation of the receptors by their endogenous ligands (OT for OTR and VP for VPRs). Data points were fitted by nonlinear regression curves (sigmoidal, slope = 1); error bars depict SEM; n = 3. For EC_50_ and E_max_ values refer to Table 1.

Fmoc-SPPS, cleavage, oxidative folding, followed by preparative C_18_-HPLC purification yielded I8-arachnotocin in >95% purity (15% overall yield) (Fig. 2). The concentration-response curves of I8-arachnotocin at the human OT/VP receptors (Fig. 3) indicated discriminatory effects in terms of E_max_ and EC_50_. At both V_1b_R and V_2_R, it was a full agonist, yet with highly disparate potencies, namely potencies of 1.2 µM (logEC_50_ = − 5.93 ± 0.15) and 34 nM (logEC_50_ − 7.47 ± 0.09) respectively. At OTR, I8-arachnotocin was a partial agonist (E_max_ = 62%) with an EC_50_ of 790 nM (logEC_50_ = − 6.11 ± 0.20) (Fig. 3). At all three receptors, I8-arachnotocin was less potent compared to the respective endogenous peptide (OTR 65-fold, V_1b_R 750-fold, V_2_R 5,000-fold). No activation of V_1a_R by I8-arachnotocin was observed for concentrations up to 10 µM (Table 1).

**Table 1:** Potency and efficacy (G protein-mediated) of I8-arachnotocin at human receptors.

	I8-arachnotocin			VP/OT^&^		
	EC_50_	pEC_50_	E_max_^#^	EC_50_	pEC_50_	E_max_^#^
V_2_R	34 nM	−7.47 ± 0.09	99 ± 3%	6.7 pM	−11.17 ± 0.10	100%
V_1b_R	1.2 µM	−5.93 ± 0.15	104 ± 8%	1.6 nM	−8.80 ± 0.09	100%
V_1a_R	antagonist pA2 of 6.253 (~558 nM)			0.56 nM	−9.25 ± 0.07	100%
OTR	790 nM	−6.11 ± 0.20	62 ± 6%	12 nM	−7.91 ± 0.09	100%

^#^EC_50_ is given in nM or µM (as indicated) and as pEC_50_ as logEC_50_ ± SEM.

^&^controls were VP at V_2_R, V_1a_R, V_1b_R and OT at OTR.

### I8-arachnotocin is a competitive antagonist at human V_1a_R

**Figure 4:**
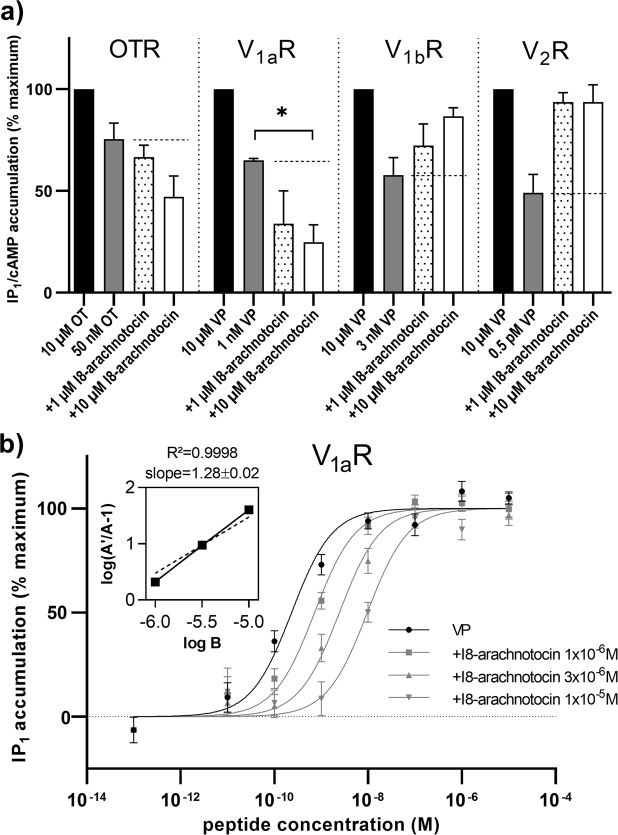
I8-arachnotocin is a competitive antagonist at V_1a_R. **(a)** Accumulation of second messengers (IP_1_ and cAMP) with partial activating concentrations of endogenous ligands (50 nM OT at OTR; 1 nM VP at V_1a_R; 3 nM VP at V_1b_R; 0.5 pM at V_2_R) in the absence and presence of 1 or 10 µM I8-arachnotocin in comparison to a saturating concentration of endogenous ligand (10 µM). All data were normalized to second messenger accumulation above baseline (0%) and maximum (100%) activity of the endogenous ligand. The dashed line depicts IP_1_/cAMP accumulation in the absence of I8-arachnotocin. Error bars depict SEM. n = 3, except n = 2 for V_1a_R (error bars depict SD). The asterisk (*) indicates significance in Student’s t-test (p = 0.0226). **(b)** Accumulation of IP_1_ by stimulation of human V_1a_R with VP (10 pM – 10 µM) alone or in the presence of 1, 3 or 10 µM I8-arachnotocin. Receptor activation was normalized to the accumulation of IP_1_ above baseline. Data points were normalized to the maximum (100%) and minimum (0%) response generated by VP. Error bars depict SEM; n = 3. Insert: Schild regression analysis: A = EC_50_ of VP in presence of I8-arachnotocin; A′ = EC_50_ of VP; B = logarithm of I8-arachnotocin concentration; Schild slope 1.28 ± 0.02 (SEM), R^2^ = 0.9998. The dotted line represents a reference line with a slope of 1; n = 3.

Based on the lack of V_1a_R activation, we performed an antagonist screen of I8-arachnotocin across all four receptors (Fig. 4a). Receptor stimulation with VP (0.55 nM) in the presence of 1 and 10 µM I8-arachnotocin, yielded lower IP_1_ levels than VP alone (Student’s t-test, p = 0.0226). The concentration-response curves of VP on V_1a_R in the absence and presence of I8-arachnotocin (1 µM, 3 µM, 10 µM) indicated an I8-arachnotocin-mediated dextral shift of the potency proportional to its concentration, without affecting E_max_, typical for a competitive antagonist (Fig. 4b). The dextral shift was evaluated *via* Schild regression analysis^26^ yielding a linear regression slope of 1.28 ± 0.02 and a pA2 of 6.253 (~558 nM), thus demonstrating that I8-arachnotocin is a competitive antagonist of the V_1a_R.

### I8-arachnotocin does not induce β-arrestin-1 or -2 recruitment at V_2_R

**Figure 5:**
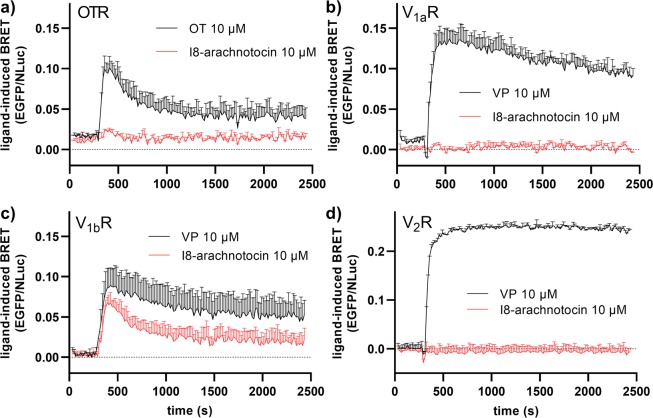
Kinetics of OT/VP- and I8-arachnotocin-induced β-arrestin-2 recruitment at human OT/VP receptors. BRET was monitored between Nano-luciferase (Nluc) and EGFP introduced at the C-terminus of (a) OTR, (b) V_1a_R, (c) V_1b_R and (d) V_2_R (EGFP-OT/VP receptors) and the β-arrestin-2 (β-arrestin-2-Nluc). HEK293 cells co-expressing EGFP-OT/VP receptors and β-arrestin-2-Nluc were stimulated by 10 µM of VP, OT, and I8-arachnotocin respectively, 5 min after addition of the luciferase substrate (furimazine). The results are shown as differences in the BRET signals in the presence of ligands and are expressed as the mean value ± SD; n = 2.

With ligand bias becoming more relevant in understanding (patho)physiological responses, we also measured I8-arachnotocin-induced β-arrestin-2 recruitment. A concentration of 10 µM of the endogenous ligand induced rapid recruitment of β-arrestin-2 across all human OT/VP receptors, as judged by the increasing BRET signal from basal to maximum in 50–100 s. However, this effect was absent upon stimulation with 10 µM of I8-arachnotocin at OTR-, V_1a_R- and V_2_R-expressing cells (Fig. 5).

**Figure 6:**
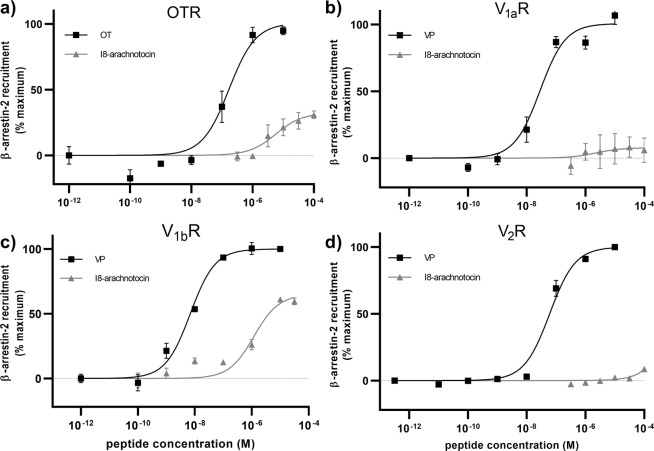
Differences between I8-arachnotocin- *vs*. VP-induced β-arrestin-2 recruitment at the human V_2_R. Concentration response curves of I8-arachnotocin and OT/VP at **(a)** OTR, **(b)** V_1a_R, **(c)** V_1b_R and **(d)** V_2_R using HEK 293 cells co-expressing EGFP-tagged receptors and β-arrestin-2-Nluc. Cells were pretreated with furimazine and measurements were taken 5 min after addition of ligands. Ligand-induced BRET was calculated as: (emission EGFP_ligand_/emission NLuc_ligand_) − (emission EGFP_HBSS_/emission NLuc_HBSS_). Results were normalized to β-arrestin-2 recruitment in response to OT/VP. Data points were fitted by nonlinear regression curves (sigmoidal, slope = 1); error bars indicate SEM; n = 3.

To further explore this effect, we measured concentration-response curves of β-arrestin-2 recruitment across all four receptors (Fig. 6, Table 2). At the V_2_R, I8-arachnotocin did not recruit β-arrestin-2 up to a concentration of 100 µM, in contrast to VP which recruited β-arrestin-2 with an EC_50_ of 60 nM (logEC_50_ = − 7.22 ± 0.07). At the V_1b_R, I8-arachnotocin recruited β-arrestin-2 (E_max_ = 65%) with an EC_50_ of 1.2 µM (logEC_50_ = − 5.92 ± 0.14), compared to VP, which recruited β-arrestin-2 with an EC_50_ of 6.9 nM (logEC_50_ = − 8.16 ± 0.09). At the OTR, I8-arachnotocin recruited β-arrestin-2 with low efficacy (E_max_ = 32%) with an EC_50_ of 5.7 µM (logEC_50_ = − 5.24 ± 0.26), compared to OT which recruited β-arrestin-2 with an EC_50_ of 170 nM (logEC_50_ = − 6.77 ± 0.15). At the V_1a_R, no I8-arachnotocin-induced β-arrestin-2 recruitment was detected at a concentration up to 100 µM, in contrast to VP-induced β-arrestin-2 recruitment with an EC_50_ of 28 nM (logEC_50_ = –7.55 ± 0.12); this aligned with our findings of I8-arachnotocin being a competitive antagonist at this receptor subtype. We furthermore quantified bias on OTR and V_1b_R following the method outlined by Kenakin^27^, but did not detect differences compared to OT or VP.

**Table 2:** Potency and efficacy (β-arrestin recruitment) of I8-arachnotocin at human receptors.

	I8-arachnotocin			OT/VP^&^		
	EC_50_	pEC_50_	E_max_^#^	EC_50_	pEC_50_	E_max_^#^
V_2_R_β-arr1_	n.d.			56 nM	−7.25 ± 0.07	100%
V_2_R_β-arr2_	n.d.			60 nM	−7.22 ± 0.07	100%
V_1b_R	1.2 µM	−5.92 ± 0.14	65 ± 5%	6.9 nM	−8.16 ± 0.09	100%
V_1a_R	n.d.			28 nM	−7.55 ± 0.12	100%
OTR	5.7 µM	−5.24 ± 0.26	32 ± 5%	170 nM	−6.77 ± 0.15	100%

^#^EC_50_ is given in nM or µM (as indicated) and as pEC_50_ as logEC_50_ ± SEM.

^&^controls were VP at V_2_R, V_1a_R, V_1b_R and OT at OTR; n.d., not detectable; β-arr, β-arrestin.

**Figure 7:**
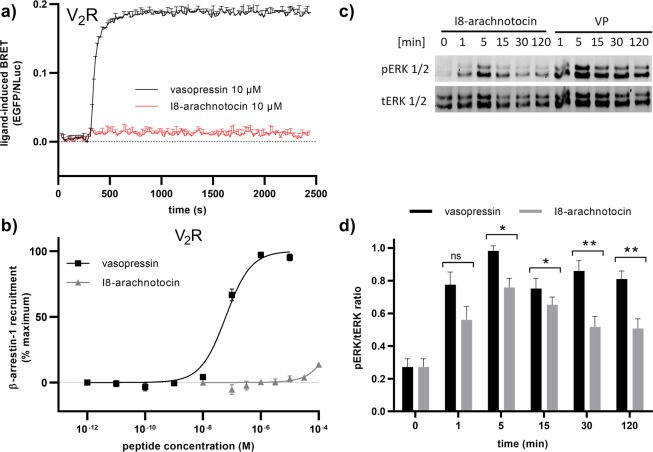
Characterization of β-arrestin-1 recruitment and ERK 1/2 phosphorylation induced by I8-arachnotocin *vs*. VP at the human V_2_R. **(a)** Kinetic profile of VP- and I8-arachnotocin-mediated β-arrestin-1 recruitment in HEK293 cells co-expressing EGFP-V_2_R and β-arrestin-1-Nluc. Cells were stimulated by 10 µM of VP or I8-arachnotocin, respectively, 5 min after addition of the luciferase substrate (furimazine). The results are shown as differences in the BRET signals in the presence of ligands and are expressed as the mean value ± SD; n = 2. **(b)** Concentration-response curves of VP and I8-arachnotocin at V_2_R using HEK 293 cells co-expressing EGFP-tagged V_2_R and β-arrestin-1-Nluc. Cells were pretreated with furimazine and measurements were taken 5 min after addition of ligands. Ligand-induced BRET was calculated as: (emission EGFP_ligand_/emission NLuc_ligand_) − (emission EGFP_HBSS_/emission NLuc_HBSS_). Results were normalized to β-arrestin-1 recruitment in response to VP. Data points were fitted by nonlinear regression curves (sigmoidal, slope = 1); error bars indicate SEM; n = 3. **(c)** Representative Western blot images of ERK 1/2 phosphorylation by stimulation of V_2_R with I8-arachnotocin *vs*. VP and **(d)** quantification of I8-arachnotocin- and VP-induced phosphorylated ERK 1/2 (pERK) relative to total ERK 1/2 (tERK) from four independent experiments (±SEM). Cells were transiently transfected with EGFP-V_2_R encoding plasmid and treated with 1 µM I8-arachnotocin or 1 µM VP at 37 °C for indicated time periods. Immunoblots were prepared from the same membranes using the same exposure method. Regions of interest were cropped from the full image (see Supplementary Information). Statistical significance was determined by Student’s t test (*P < 0.05; **P < 0.01; ns non-significant).

Since recent studies uncovered an overlapping role of β-arrestin-1 and β-arrestin-2 with regard to V_2_R-dependent agonist-induced endocytosis and ERK activation^28^, we probed whether I8-arachnotocin is capable of recruiting β-arrestin-1 in a BRET-based assay. In comparison to VP (10 µM), which robustly induced β-arrestin-1 recruitment, I8-arachnotocin (10 µM) also failed to recruit β-arrestin-1 in a time-dependent manner (Fig. 7a). Moreover, VP treatment resulted in a concentration-dependent β-arrestin-1 recruitment with an EC_50_ of 56 nM (logEC_50_ = –7.25 ± 0.07), but receptor stimulation with I8-arachnotocin exhibited no β-arrestin-1 recruitment up to 100 µM (Fig. 7b). Overall, data obtained by BRET studies clearly demonstrate that modulation of the V_2_R with I8-arachnotocin results in neither recruitment of β-arrestin-1 nor β-arrestin-2, thereby confirming bias of this ligand towards G protein-coupling.

### I8-arachnotocin modulates V_2_R-mediated ERK signaling

Having established that I8-arachnotocin is a G protein-biased peptide ligand, we examined its ability to modulate V_2_R-mediated ERK 1/2 phosphorylation. Previous studies reported that V_2_R activates ERK by two different pathways hypothesized to be dependent on either Gα_s_ or β-arrestin signaling with distinct temporal patterns; the early phase (<5 min) being mediated by the G protein-dependent pathway, while the later phase (>10 min) being β-arrestin-2 dependent^29^. More recently, a study utilizing a combination of CRISPR/Cas9 genome-editing and pharmaceutical inhibition to generate HEK293 cells with ‘zero functional G’ indicated that ERK signaling mediated by V_2_R may still be dependent upon G protein even at later time points at which it may also be β-arrestin-dependent^30^. HEK293 cells transiently transfected to express the human V_2_R were stimulated with 1 µM I8-arachnotocin and 1 µM vasopressin for time periods between 1 min and 2 h. The immunoblotting data demonstrate that the late phase of V_2_R-dependent ERK 1/2 activation induced by I8-arachnotocin is significantly different from the late phase of VP-stimulated ERK 1/2 activation (30–120 min; Fig. 7c,d). While both peptides provoked an early and rapid ERK 1/2 phosphorylation peaking at 5 min, I8-arachnotocin-stimulated ERK 1/2 phosphorylation decreased over time in contrast to VP-elicited ERK 1/2 activation that resulted in a more sustained and prolonged ERK 1/2 activation (Fig. 7c,d). These data further support that I8-arachnotocin is a biased peptide ligand that modulates ERK 1/2 activity by preferentially activating the early phase G protein-dependent pathway at the plasma membrane over the late phase pathway that is β-arrestin-dependent.

## Discussion

Peptides are gaining momentum in the drug development field, due to their (i) ability to interact with proteins on a large surface and (ii) structural and chiral complexity, which allows for improved discrimination between highly homologous targets as compared to small molecules^14^. This is particularly the case for the OT/VP signaling system, where multiple small molecule drugs failed due to selectivity issues and the majority of approved therapeutics are peptide drugs^15,31^. The study of the complex signaling pathways of the widely-distributed and fundamental OT/VP signaling system remains however challenging due to the limited availability of pharmacological probes^14,15,18^. Differentiating between signaling events that occur pre or post β-arrestin recruitment has become an important focus^32–34^ for studies looking at understanding the (patho)physiological roles of β-arrestin-mediated receptor internalization, desensitization and trafficking^35,36^. In addition, β-arrestin-dependent signaling has been linked to chronic stress-evoked melanoma metastasis *via* OTR^37^, increased neonatal rat cardiac fibroblast proliferation *via* V_1a_R^38^, morphine tolerance *via* V_1b_R^39^ and sustained non-canonical signaling after receptor internalization *via* V_2_R, resulting in strong antidiuretic and anti-natriuretic effects^40^. Biased ligands such as I8-arachnotocin discovered in this work are thus important tools to advance our understanding in these areas of interest.

By utilizing a drug discovery strategy^14,41^ on the synthesis of evolutionarily-conserved, yet distinct, peptides, we were able to bypass the time- and resource-consuming fractionation, isolation and identification steps associated with the discovery of plant- or venom-derived compounds such as kalata B7^23^, inotocin^19^ and conopressin T^22^. This strategy led us to explore the vast and untapped arthropod kingdom and resulted in the discovery and pharmacological characterization of I8-arachnotocin.

I8-arachnotocin activated the Gα_s_ (cAMP) pathway, inducing ERK 1/2 phosphorylation without detectable recruitment of β-arrestin-1 or -2 at V_2_R, despite the capability of this receptor to form stable and strong interactions with β-arrestins^42^. These findings are consistent with the observation that I8-arachnotocin induced substantially lower levels of ERK 1/2 phosphorylation at later time points compared to VP, leading us to conclude that I8-arachnotocin displays a clear bias away from β-arrestin-dependent signaling at V_2_R. We are not aware of another V_2_R ligand capable of selectively activating the non-β-arrestin-dependent (EC_50_ = 50 nM) *vs*. the β-arrestin-dependent (EC_50_ > 100 µM) signaling pathway (>2,000-fold difference). Such biased ligands are highly sought-after for research tools that allow for the discrimination between multiple active conformations of GPCRs^32,33^. Since the pharmacology of the β-arrestin-1 and -2 pathways in respect to the OT and VP receptors is not fully elucidated yet^43,44^, I8-arachnotocin represents a valuable first probe to advance our understanding of this pathway at the human V_2_R.

Our data suggest that I8-arachnotocin can only effectively recruit β-arrestin-2 at the V_1b_R (E_max_ = 65%). To try to understand the structural differences resulting in bias between the four receptors, we compared the binding site residues of the V_1b_R *vs*. the OTR, V_1a_R and V_2_R; there are two positions that differ in these receptors, i.e. position 7.30 (Thr *vs*. Glu) and 7.42 (Asn *vs*. Ser)^19^. Although there are only a limited number of reports dealing with structural changes in GPCRs responsible for arrestin recruitment, it has been suggested for instance that residues in TM6 and TM7 are important for pathway selectivity^45,46^. Hence, the identified residues in positions 7.30 and 7.42 of the ligand binding pocket of OT/VP receptors, could contribute to the observed bias of the bound I8-arachnotocin ligand, by altering the interaction of the receptor C-tail with the N-terminal domain of arrestin^47^. However, this remains speculative until future studies reveal further details.

Overall, GPCRs are prime drug targets^48^ and the vast chemical diversity of nature will continue to deliver novel pharmacological and therapeutic leads, particularly with technological advances that accelerate the drug discovery pipeline^49^. This work follows this innovative trend by exploiting the evolutionary conservation and ubiquity of neuropeptides across the animal kingdom^50,51^. In particular, it highlights the abundance of neuropeptides in arthropods: e.g., there are >50–150 neuropeptides reported in the model species *Tribolium castaneum*^52^, *Nasonia vitripennis*^53^, *Apis mellifera*^54^ and *Drosophila melanogaster*^55^. We thus argue that arthropods represent a novel, vast and untapped source for the discovery of pharmacological probes and potential therapeutic leads for a broad range of signaling systems.

## Supplementary information

Supplementary Information
