# Supplementary figures and images for: I8-arachnotocin–an arthropod-derived G protein-biased ligand of the human vasopressin V2 receptor
Source: Sci Rep. 2019 Dec 17;9:19295. doi: 10.1038/s41598-019-55675-w (PMC6917733; doi:10.1038/s41598-019-55675-w)

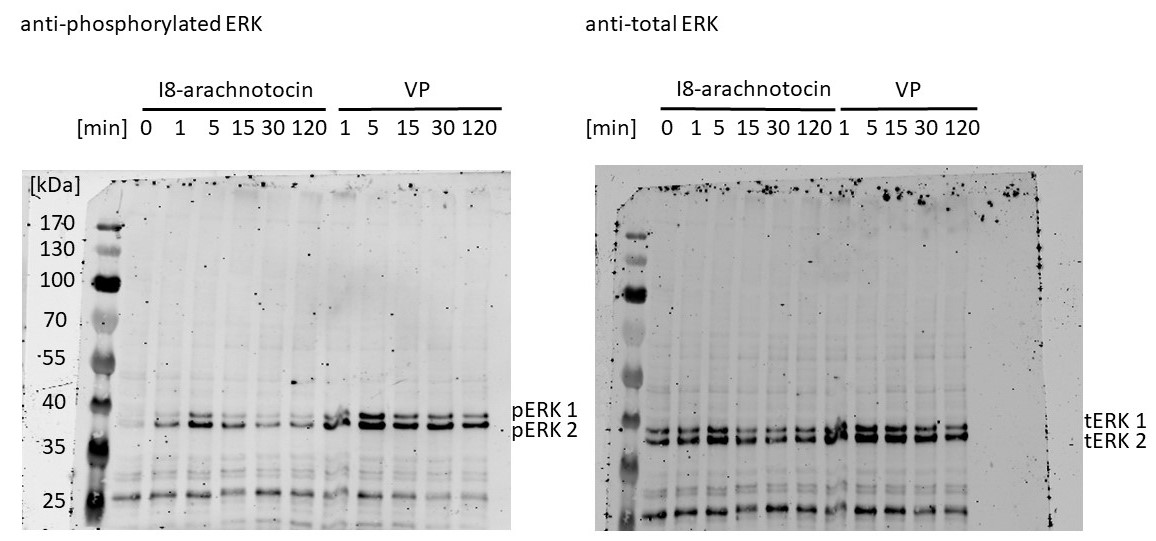

Supplement: Supplementary file 1 — Supplementary Information [file 41598_2019_55675_MOESM1_ESM.jpg]
